# Supplementary material for: Temporal and Spatial Gene Expression Profile of Stroke Recovery Genes in Mice
Source: Genes (Basel). 2023 Feb 9;14(2):454. doi: 10.3390/genes14020454 (PMC9956317; doi:10.3390/genes14020454)
Supplement: Supplementary file 1 [file genes-14-00454-s001.zip › genes-2157823-supplementary.pdf]

Supplementary Materials

Table S1. Specification of forward and reverse primer pairs (Eurofins)

Name	Sequence 5′ -> 3′	Weight [g/mol]	gDNA Amplification
Adora2a For	ACTCCGGTACAATGGCTTGGTG	6766	Possibly
Adora2a Rev	CCCAGCAAATCGCAATGATGCC	6673	Possibly
Pde10a For	TACGTGGCCAAGTCTAGGAAGACG	7427	No
Pde10a Rev	TACCTCGAGGAAATCGCTCATCCC	7258	No
Drd2 For	ACCTGAAGACACCACTCAAGGG	6722	No
Drd2 Rev	ACGGTGCAGAGTTTCATGTCCTC	7031	No
Gapdh For	CATGGCCTTCCGTGTTCCTA	6035	?
Gapdh Rev	GCGGCACGTCAGATCCA	5180	?

Table S2. List of used primers from BioRad. Note that these are premixed primers and, therefore, contain forward and reverse primers

Name	Unique Assay ID	Assay Design
Atrx	qMmuCED0048169	Exonic
Lingo1	qMmuCED0026351	Exonic
Bdnf	qmMuCED0048169	Exonic

Table S3. List of Primer efficiencies.

Primer	Efficiency
Adora2	1.99
Pde10a	1.95
Drd2	2.07
Lingo1	1.90
Atrx	1.94
Bdnf	1.84
Nos1	1.80
Gapdh	1.97

Table S4. The recovery rate of stroke mice calculated from grid walk (footfault) and rotating beam (speed and hindlimb drop) was via linear regression between days 3 to 14 post stroke (P3-P14) (left) and P3 to P7 to P14 (right). The median results of single tests were taken for the differentiation between good and poorly recovered mice. Medians P3-P14: Footfaults -0.25, RB Speed 0.73, HL drop -0.6; Medians P3-P7-P14: Footfaulta -0.40, RB Speed 0.80, HL drop -0.6. Grey filling indicated poorly recovered mice.

	**P3 - P14**				**P3-P7-P14**		
Mouse ID	**Footfaults**	**RB Speed**	**HL Drop**	Mouse ID	**Footfaults**	**RB Speed**	**HL Drop**
T3_3_1	−0.15	0,81	−0.05	T3_3_1	−0.19	0,82	−0.07
T3_3_2	0,30	0,49	−0.02	T3_3_2	0,31	0,48	−0.04
T3_4_2	−0.53	1,00	0,05	T3_4_2	−0.51	0,96	0,00
T3_4_3	−1.14	0,54	−0.01	T3_4_3	−1.13	0,61	0,00
T3_4_4	−0.61	0,49	−0.16	T3_4_4	−0.70	0,51	−0.16
T3_4_5	−0.45	0,73	0,01	T3_4_5	−0.49	0,80	0,00
T3_5_2	−0.30	1,11	−0.07	T3_5_2	−0.34	0,97	−0.06
T3_6_1	−0.25	1,46	0,02	T3_6_1	−0.25	1,46	0,02
T3_7_2	0,08	1,29	−0.30	T3_7_2	0,08	1,29	−0.30
T3_8_1	0,76	0,96	−0.10	T3_8_1	0,76	0,96	−0.10
T3_8_3	0,24	0,45	−0.39	T3_8_3	0,24	0,45	−0.39
T3_8_4	−0.45	0,81	−0.07	T3_8_4	−0.45	0,81	−0.07
T3_8_5	−0.23	−0.14	−0.05	T3_8_5	−0.23	−0.14	−0.05
T3_9_1	−1.00	0,42	−0.70	T3_9_1	−0.92	0,38	−0.64
T3_9_3	0,15	0,44	−0.75	T3_9_3	0,09	0,44	−0.67
T3_9_4	−0.58	0,29	−0.11	T3_9_4	−0.54	0,23	−0.10
T3_10_2	0,20	0,84	−0.11	T3_10_2	0,29	0,80	−0.11
T3_10_3	−0.45	−0.03	−0.02	T3_10_3	−0.40	−0.02	−0.02
T3_12_1		1,13	0,00	T3_12_1	−0.42	1,06	0,00
T3_12_2		1.43	0.00	T3_12_2	0.42	1.28	0.00
T3_12_4		0.71	−0.05	T3_12_4	−0.84	0.7	−0.03
T3_13_4		0.39	0.05	T3_13_4	0.42	0.29	0.05
T3_14_1		0.99	−0.16	T3_14_1	−1.46	0.86	−0.15
T3_14_3		0,70	0,00	T3_14_3	−0.84	0,65	0,00
T3_14_5		1,24	−0.09	T3_14_5	−0.83	1,21	−0.08

Table S5. Mice can be grouped into good recovery, neutral, or poor recovery mice according to their results in grid walk and rotating beam tests

**Good Recovery**		**Neutral**	**Poor Recovery**	
PB_T3_4_2	PB_T3_4_4	PB_T3_3_1	PB_T3_3_3	PB_T3_4_3
PB_T3_5_2	PB_T3_7_2	PB_T3_4_5	PB_T3_8_3	PB_T3_8_5
PB_T3_8_1	PB_T3_8_4	PB_T3_6_1	PB_T3_9_3	PB_T3_10_3
PB_T3_9_1	PB_T3_9_4		PB_T3_12_4	PB_T3_13_4
PB_T3_10_2	PB_T3_12_1		PB_T3_14_3	
PB_T3_12_2	PB_T3_14_1			
PB_T3_14_5				

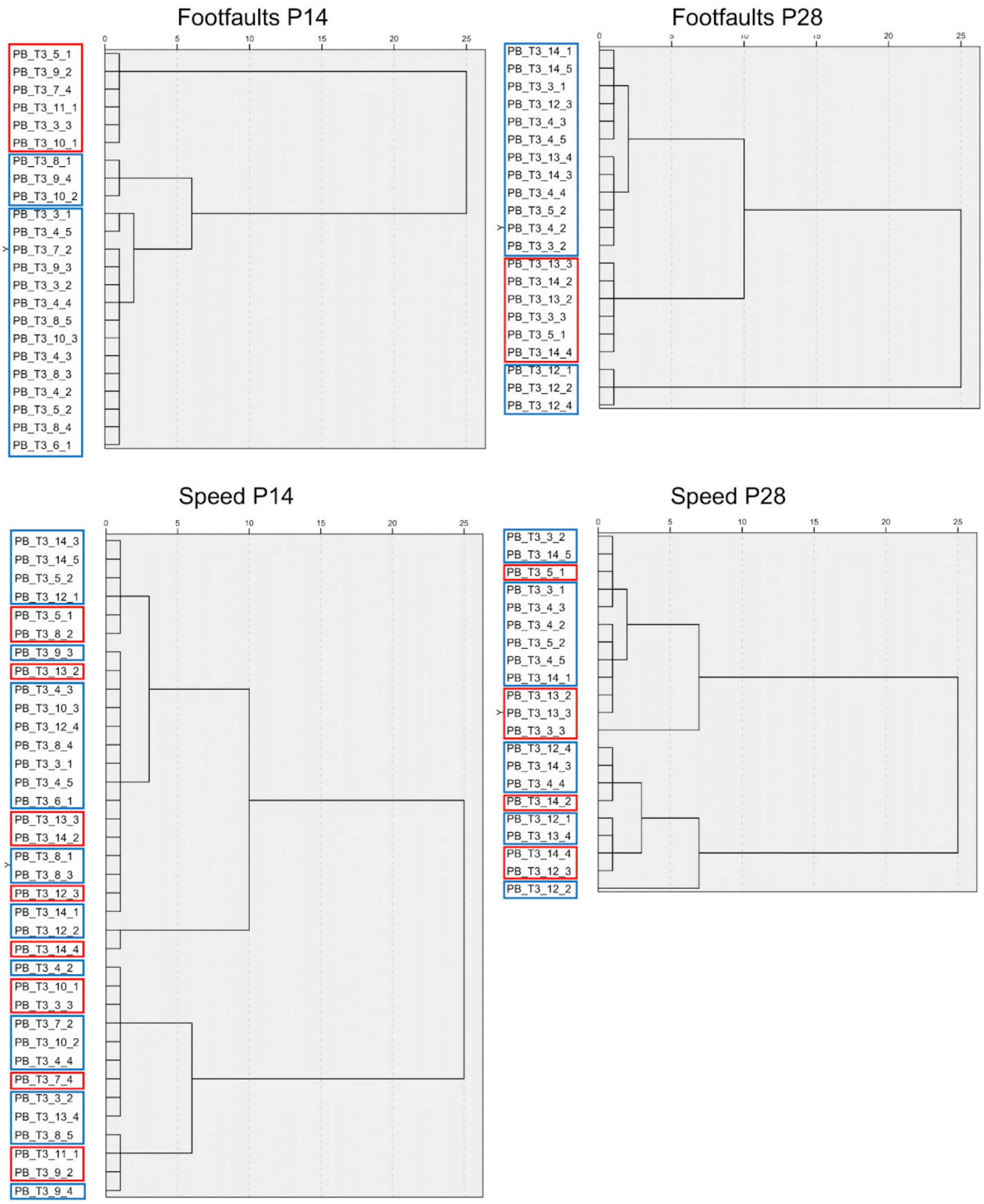

Figure S1: Dendrograms of cluster analysis by SPSS from days 14 and 28 after stroke for foot faults at the grid walk test and speed of the rotating beam test. Red and blue frames mark sham and stroke animals, respectively. The x-axis shows the distance between the clusters with a range of 1-25, whereby at 1, every individual data point is a cluster. The y-axis shows the data points.

Key findings (comparison good/poor vs. sham mice, Figure S2)

MOp: showed higher expressions of *Adora2a*, *Pde10a,* and *Drd2* at P14/56, whereas *Lingo1* and *BDNF* had lower expressions at P14 and *Atrx* lower expressions at P14 and higher expressions at P56.

SSp: expression of *Adora2a*, *Pde10a,* and *Drd2* in the SSp increased at P28 and P56 and was higher in the good recovery group, whereas expression of *Lingo1*, *BDNF,* and *Atrx* showed unclear or no change in expression over all time points.

Str: Well- recovered mice showed a higher expression of *Adora2a*, *Pde10a,* and *Drd2* at 28 days post-stroke in the il Str, while *Lingo1* and *BDNF* had increased expressions at P56, and *Atrx* showed unclear or no change.

TH: *Adora2a* and *Pde10a* in the TH were more expressed at P56, mainly in well-recovered mice, while *Drd2* was downregulated at 28 days post-stroke and upregulated at P56. *Lingo1*, *BDNF,* and *Atrx* showed unclear or no change.

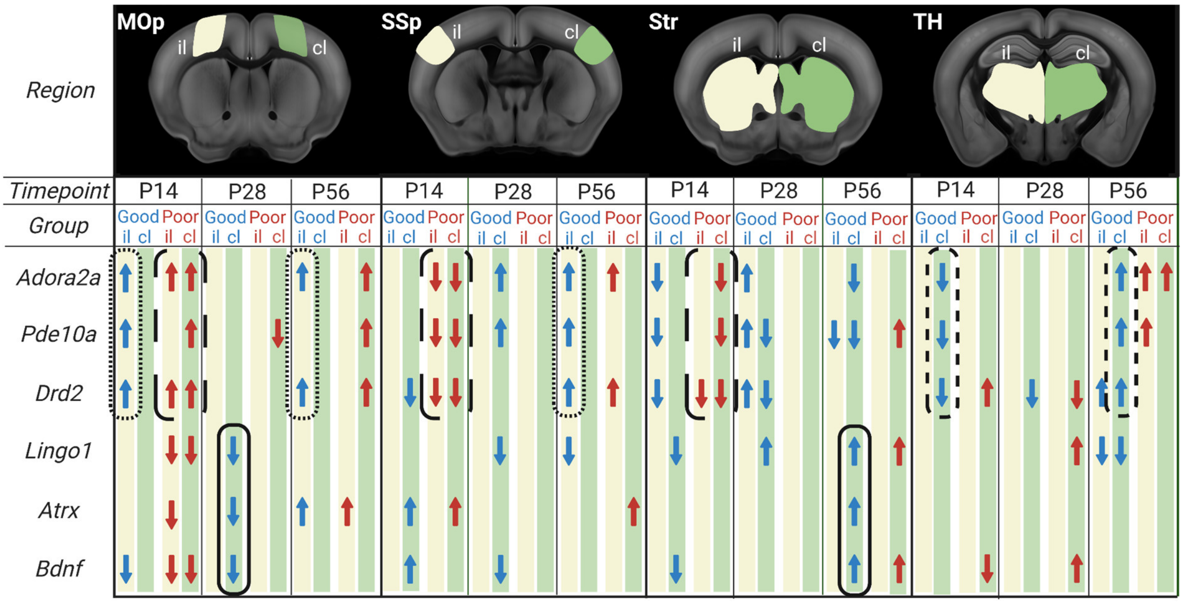

Figure S2. Gene expression summary at 14, 28, and 56 days post-stroke compared to sham. Gene expression analysis results of well (blue) and poorly (red) recovered mice, compared to sham, divided into brain parts, hemisphere (yellow and green), and days post-stroke (P14, P28, P56). Patterns were marked with different dotted lines. Figure created with BioRender.com (accessed on 28 December 2022).
